# An extensive quantitative analysis of the effects of errors in beat-to-beat intervals on all commonly used HRV parameters

**DOI:** 10.1038/s41598-023-50701-4

**Published:** 2024-01-30

**Authors:** Maurice Rohr, Mika Tarvainen, Seyedsadra Miri, Gökhan Güney, Antti Vehkaoja, Christoph Hoog Antink

**Affiliations:** 1 AI Systems in Medicine, Technical University of Darmstadt, 64283 Darmstadt, Germany; 2 Department of Technical Physics, University of Eastern Finland, 70211 Kuopio, Finland; 3 Department of Clinical Physiology and Nuclear Medicine, Kuopio University Hospital, 70211 Kuopio, Finland; 4 Faculty of Medicine and Health Technology, Tampere University, 33720 Tampere, Finland; 5 Finnish Cardiovascular Research Center, 33720 Tampere, Finland

**Keywords:** Biomarkers, Cardiology, Biomedical engineering

## Abstract

Heart rate variability (HRV) analysis is often used to estimate human health and fitness status. More specifically, a range of parameters that express the variability in beat-to-beat intervals are calculated from electrocardiogram beat detections. Since beat detection may yield erroneous interval data, these errors travel through the processing chain and may result in misleading parameter values that can lead to incorrect conclusions. In this study, we utilized Monte Carlo simulation on real data, Kolmogorov–Smirnov tests and Bland–Altman analysis to carry out extensive analysis of the noise sensitivity of different HRV parameters. The used noise models consider Gaussian and student-t distributed noise. As a result we observed that commonly used HRV parameters (e.g. pNN50 and LF/HF ratio) are especially sensitive to noise and that all parameters show biases to some extent. We conclude that researchers should be careful when reporting different HRV parameters, consider the distributions in addition to mean values, and consider reference data if applicable. The analysis of HRV parameter sensitivity to noise and resulting biases presented in this work generalizes over a wide population and can serve as a reference and thus provide a basis for the decision about which HRV parameters to choose under similar conditions.

## Introduction

Heart rate variability (HRV) is an indirect indicator of the state of the autonomic nervous system (ANS). HRV has been increasingly used for example in exercise recovery assessment^1^ and in wellness applications for assessing sleep quality^2^. Many research articles have also proposed its use in clinical applications. According to a recent review on HRV applications in the medical domain by Faust et al.^3^ cardiology is the most studied application area for HRV followed by mental health and sleep physiology. In cardiology, HRV is widely used for detecting cardiac arrhythmias. However, in arrhythmia applications, the purpose is not to obtain information on the state of ANS but rather to detect and analyze ectopic heartbeats originating from elsewhere in the heart rather than the sinoatrial node. Cardiological HRV applications assessing ANS include early detection of surgical stress^4^, detection and monitoring of heart failure, risk prediction for sudden cardiac death, and several others^3^. HRV has been proposed as a clinical tool for routine risk stratification in myocardial infarction patients^5^, but generally more trials and research are needed for a wider application in clinical practice.

One of the most widely used HRV parameters is the standard deviation of beat-to-beat intervals (SDNN)^1^. It reflects the overall activity of ANS regulation and it is considered as the gold standard for evaluating cardiac risk^6^. It has been found to be directly affected by myocardial infarction (MI)^7^, autonomic dysfunction^8^ and mental conditions such as depression and anxiety^9^.

In several applications, it is important to detect even small changes in the HRV parameter values. However, the parameters are sensitive to the uncertainty or errors in the heartbeat intervals. Given that different HRV parameters carry redundant information about the ANS, it would be interesting to assess the differences in their sensitivity to the heartbeat interval errors. This would help to select those HRV parameters that are less sensitive to the errors but still provide relevant information.

While the ECG-based RR-interval tachogram is the gold standard data for HRV estimation, using pulse intervals measured with photoplethysmography (PPG) for HRV estimation has recently received a lot of interest. PPG-based HRV is usually referred to as pulse rate variability (PRV) to highlight the inevitably higher uncertainty in the heartbeat intervals as well as the fundamental difference caused by the variations in pulse arrival time due to changes in blood pressure^10^. In PRV analysis, the robustness of HRV parameters to uncertainty in beat-to-beat intervals is therefore even more important.

Most of the earlier studies on HRV sensitivity to heartbeat interval uncertainty have focused on evaluating the effect of ECG sampling rate and resulting uncertainty in the temporal location of the R-peak as well as on the sporadic errors in R-peak detection, missing R-peaks, and the effect of beat replacement^11,12^. Usually, the studies have focused on evaluating the effects in a few most commonly used HRV parameters^13,14^. Petelczyc et al.^15^ performed one of the most thorough analysis using Monte Carlo simulation for comparing the sensitivity of various HRV parameters on the ECG sampling frequency and QRS complex detection and estimated the effect of RR-interval errors in ten commonly used HRV parameters. They found that the most sensitive parameter was pNN50. On the other hand, short and long-term slopes, $\alpha _1$ and $\alpha _2$ of the detrended fluctuation analysis (DFA) were found to be the least sensitive to RR-interval errors. In the present work, we perform extensive Monte Carlos simulations in which we artificially introduce error in real-world RR-interval data and analyze how this noise is reflected in 34 HRV parameters calculated in 5-min segments. In our simulations, we vary both the distribution of the noise (uniform, Gaussian, t-distributed) as well as its standard deviation between 1 to 10 ms to approximate interval errors for example due to low sampling rate in ECG and average errors seen in PPG^16^. We present our results both quantitatively as well as qualitatively and perform statistical tests to find significant differences. We assess the consistency of the effect of the noise distribution and evaluate, which HRV parameters show consistent bias under the presence of RR-interval uncertainty and which are more and which are less affected by it.

The main contribution of this study is an overview over the error distributions of an extensive selection of different HRV parameters. We quantify their sensitivity to noise and show that most parameters show systematic biases. Based on our results, we argue that LF/HF ratio and pNN50 should be used with caution and that researchers should generally consider the distributions of parameter values instead of only computing mean and median values.

## Material and methods

In order to obtain realistic beat-to-beat intervals from healthy subjects, the “Autonomic Aging Dataset” was used^17^, which is a publicly available dataset within the PhysioNet Database^18^. It contains recordings of 1121 healthy volunteers of approximately 8–40 min duration. It is divided into six age groups (below 30, 30–39, 40–49, 50–59, 60–69, above 69 years) with 670 participants being female and 433 male. For each subject, an ECG signal (lead II) and a continuous non-invasive blood pressure signal is available. The ECG is recorded at 1000 Hz either by an MP150 (ECG100C, BIOPAC systems inc., Golata, CA, USA) or Task Force Monitor system (CNSystems Medizintechnik GmbH, Graz, AUT). All subjects were screened rigorously and remained in a resting state sinus rhythm. In order to obtain clean and representative heartbeat interval data and heartbeat annotations, we extracted the heartbeat locations from the ECG using a Pan-Tompkins based QRS detector^19^ and employed a validated beat correction algorithm^20^. Instead of using the corrected beats, segments containing overwritten missed or erroneous beats were discarded, reducing the number of subjects with more than 5 min of recording to 971. The processed dataset contains a total of 989,399 automatically validated heart beats.

In our simulation, we created a representative sample ${\mathcal {S}}$ of $n=15{,}000$ 5-min segments from the processed dataset. *n* was chosen as an upper bound for the convergence criterion for the distribution of each HRV parameter, which we defined as the point where the normalized standard error for the expectation of the HRV parameter distribution (standard error $S_e$/sample mean $\mu _s$) drops below 2%, where $S_e=1.96\sigma _s/\sqrt{n}$ and $\sigma _s$ is the sample standard deviation. Sampling was performed by first choosing one of the 971 recordings at random, selecting a starting heart beat annotation at random, and then including all beat annotations in the following 5-min window, leading to windows with partially overlapping information. The average number of heartbeats in each window was 344 with a standard deviation of 48, corresponding to an average heart rate of 69 bpm with a standard deviation of 9.6 bpm. Due to the relatively low number of sampling iterations not all subjects were represented equally by $n/971=15$ segments. Notably, the distribution of segments per age group matches the age distribution in the Autonomic Aging dataset, which is heavily tilted towards the younger groups.

### HRV calculation

HRV analyses were carried out using Kubios HRV Scientific 4.0 software (Kubios Oy, Kuopio, Finland). The pre-processing features of Kubios HRV software including noise detection, beat correction, and detrending were all disabled in order to observe the true influence of IBI errors over different HRV analysis parameters. Otherwise, the HRV parameters were derived according to the guidelines^21^. The extensive set of HRV parameters assessed in this study are described in Table 1.

**Table 1:** Descriptions of assessed time-domain, frequency-domain and nonlinear HRV parameters and related analysis settings.

Parameter	Units	Description
Time-domain HRV parameters		
Mean RR	(ms)	The mean of RR intervals
Mean HR	(bpm)	The mean heart rate (60 s/Mean RR)
SDNN	(ms)	Standard deviation of (normal-to-normal) RR intervals
RMSSD	(ms)	Square root of the mean squared differences between successive RR intervals
NN50	(beats)	Number of successive RR intervals that differ more than 50 ms
pNN50	(%)	NN50 divided by the total number of RR intervals $\times$ 100%
HRVti		HRV triangular index derived as the area of the RR interval histogram divided by the height of the histogram
TINN	(ms)	Baseline width of the RR interval histogram derived using triangular interpolation
SI		Square root of the Baevsky’s stress index^22^
DC	(ms)	Heart rate deceleration capacity (DC) derived as four-point difference from the deceleration phase^23^
DC_mod_	(ms)	Modified DC computed as a two-point difference^24^
AC	(ms)	Heart rate acceleration capacity (AC) derived as four-point difference from the acceleration phase^23^
AC_mod_	(ms)	Modified AC computed as a two-point difference^24^
Frequency-domain HRV parameters		
LF power	(ms^2^)	Absolute power of low frequency (LF: 0.04–0.15 Hz) component
HF power	(ms^2^)	Absolute power of high frequency (HF: 0.15–0.4 Hz) component
pLF power	(%)	Relative power of LF component: LF (%) = LF (ms^2^)/Total power (ms^2^) $\times$ 100%
pHF power	(%)	Relative power of HF component: HF (%) = HF (ms^2^)/Total power (ms^2^) $\times$ 100%
nLF power	(n.u.)	LF power in normalized units: LF (n.u.) = LF (ms^2^)/[Total power (ms^2^) − VLF (ms^2^)] $\times$ 100%, where VLF is the power of the very low frequency (0–0.04 Hz) component
nHF power	(n.u.)	HF power in normalized units: HF (n.u.) = HF (ms^2^)/[Total power (ms^2^) − VLF (ms^2^)] $\times$ 100%
LF/HF		Ratio between LF and HF component powers
Total power	(ms^2^)	Total spectral power
Nonlinear HRV parameters		
Poincaré SD1	(ms)	In Poincaré plot, the standard deviation perpendicular to the line-of-identity^25^
Poincaré SD2	(ms)	In Poincaré plot, the standard deviation along the line-of-identity^25^
Poincaré SD2/SD1		The ratio between SD2 and SD1
ApEn		Approximate entropy^26^
SampEn		Sample entropy^26^
DFA $\alpha _1$		In detrended fluctuation analysis (DFA), the short-term fluctuations slope^27^
DFA $\alpha _2$		In DFA, the longer-term fluctuations slope^27^
D2		Correlation dimension^28^
RPA REC	(%)	In recurrence plot analysis (RPA), recurrence rate^29^
RPA DET	(%)	In RPA, determinism^29^
RPA Lmean	(beats)	In RPA, the mean line length^29^
RPA Lmax	(beats)	In RPA, the maximum line length^29^
RPA ShanEn		In RPA, the Shannon entropy of the line lengths^29^

### Noise simulation

**Figure 1:**
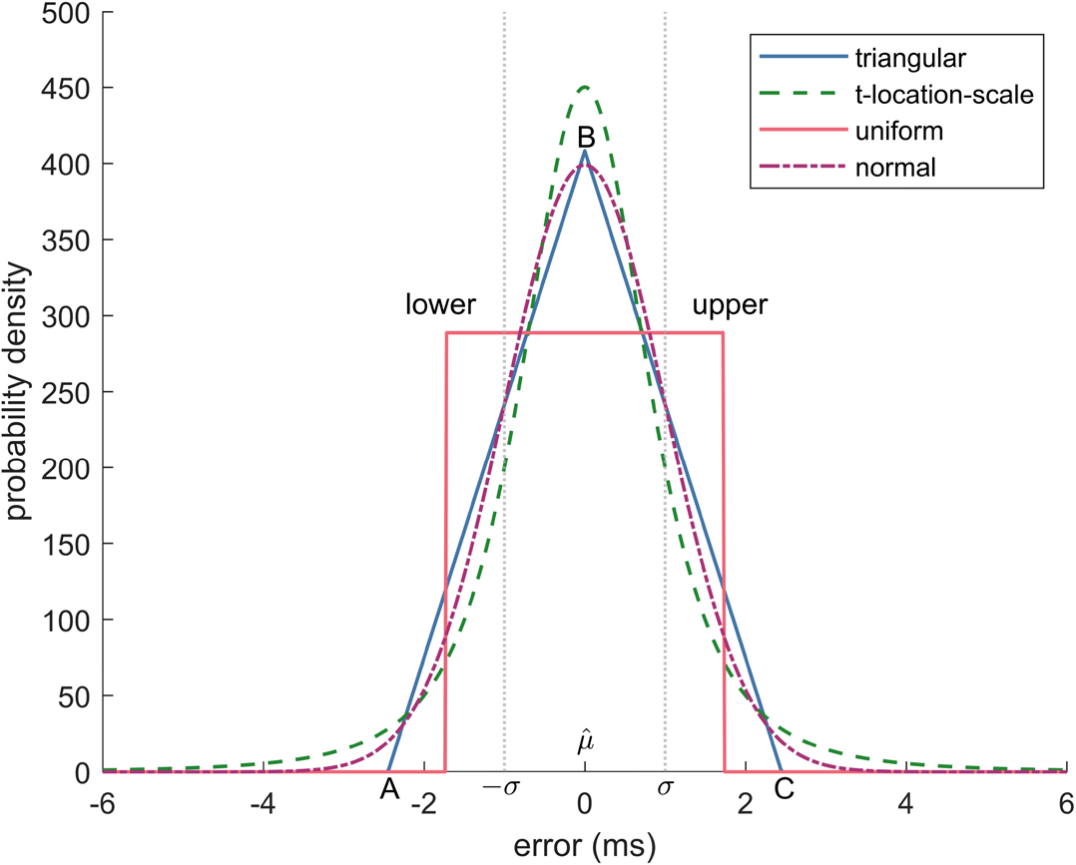
Error distributions commonly observed. All with standard deviation of 1 ms.

We added noise to the beat timings by sampling from three differently distributed random variables. We investigated 10 equally spaced noise levels with standard deviations $\sigma$ ranging from 1 to 10 ms. The distributions in Fig. 1 were parameterized to have the same standard deviations and zero-mean. The analytically derived parameters were computed as follows:

1 $$\begin{aligned} \text {Gaussian}&: {\hat{\mu }}=0,\;{\hat{\sigma }}=\sigma , \end{aligned}$$

2 $$\begin{aligned} \text {uniform}&: \text {lower}=-\sqrt{3}\sigma ,\;\text {upper}=\sqrt{3}\sigma , \end{aligned}$$

3 $$\begin{aligned} \text {triangular}&: A=-1/\sqrt{3/18}\sigma ,\; B=0,\; C=-A, \end{aligned}$$

4 $$\begin{aligned} \text {t-distribution}&: {\hat{\mu }}=0,\; {\hat{\sigma }}=\sqrt{({\hat{\nu }}-1)\sigma ^2/{\hat{\nu }}},\;{\hat{\nu }}=3 , \end{aligned}$$

where ${\hat{\nu }}$ is chosen as the minimal possible value, thus the second moment is defined to achieve very heavy tails.

The choice of distributions is motivated by errors introduced during signal processing. Due to the finite sampling frequency, peak locations in the raw data always show a uniformly distributed error^30^. Besides the sampling error, the uniform distribution is chosen because typical QRS detectors with beat corrections usually come with an upper and a lower threshold for discarding beats, which limits the possible error, but apart from that arbitrary errors are possible. Interestingly, the triangular distribution (not directly applied in this study) is generated when we compute the inter-beat intervals from uniformly distributed peaks and should therefore be considered as relevant for inter-beat interval analysis. The Gaussian distribution models the combination of multiple unknown error sources in the signal generation and processing pipeline, such as sampling-noise, quantization noise, peak-uncertainty as well as the influence of signal noise on the detection and is the most common assumption. On other accounts, Petelczyc et al.^15^ assume that the QRS detection procedure amplifies existing errors. The t-distribution is a heavy-tailed distribution and thus allows extreme errors with non-zero probability such as outliers from missed beats, extra beats, or misaligned beats due to motion artifacts or undetected ectopic beats.

### Evaluation

For every 5-min segment in ${\mathcal {S}}$, the HRV values calculated before adding noise are considered the ground truth values $x_{i,p}$, with $i \in \{1,\dots ,n\}, \, p \in \textrm{HRV}_{\mathrm{{params}}}$. After adding noise of a specific distribution and intensity, the estimated HRV value ${\hat{x}}_{i,p}$ is obtained. The difference to the ground truth that arises from the addition of noise as described above is considered the error,

5 $$\begin{aligned} e_{i,p} = {\hat{x}}_{i,p} - x_{i,p}. \end{aligned}$$

For the Bland–Altman analysis, we also need to calculate the average of the ground truth and the estimation, $a_{i,p} = ({\hat{x}}_{i,p} + x_{i,p})/2$. The systematic bias is defined as the average of the error over all $n=15000$ samples,

6 $$\begin{aligned} \Delta _p = \frac{1}{n} \sum _{i=1}^n e_{i,p}. \end{aligned}$$

In addition to the systematic bias, the mean absolute error (MAE)

7 $$\begin{aligned} \text {MAE}_p = \frac{1}{n} \sum _{i=1}^n\left| e_{i,p} \right| \end{aligned}$$

as well as the root-mean-square error (RMSE)

8 $$\begin{aligned} \text {RMSE}_p = \sqrt{ \frac{1}{n} \sum _{i=1}^n e_{i,p}^2 } \end{aligned}$$

are calculated. To further analyze the distribution of the error, the 5th and 95th percentile are calculated. The Kolmogorov–Smirnov test is performed to test whether the distributions of the HRV parameter error depend on the distribution of the noise. In our analysis we consider p-values $p<0.05$ to be statistically significant. Finally, to allow for a comparison of the noise sensitivity of the different HRV metrics, we calculate the group mean of the ground truth of all *n* windows,

9 $$\begin{aligned} {\bar{x}}_p = \frac{1}{n} \sum _{i=1}^n x_{i,p}. \end{aligned}$$

Next, we fit a linear function to determine the dependency of bias, MAE, and RMSE on $\sigma$,

10 $$\begin{aligned} \Delta _p&= \alpha _{\text {bias}}/100\% \cdot \sigma \cdot {\bar{x}}_p, \end{aligned}$$

11 $$\begin{aligned} \text {MAE}_p&= \alpha _{\text {MAE}}/100\% \cdot \sigma \cdot {\bar{x}}_p, \end{aligned}$$

12 $$\begin{aligned} \text {RMSE}_p&= \alpha _ { \text {RMSE}}/100\% \cdot \sigma \cdot {\bar{x}}_p. \end{aligned}$$

## Results

### Kolmogorov–Smirnov-test

**Figure 2:**
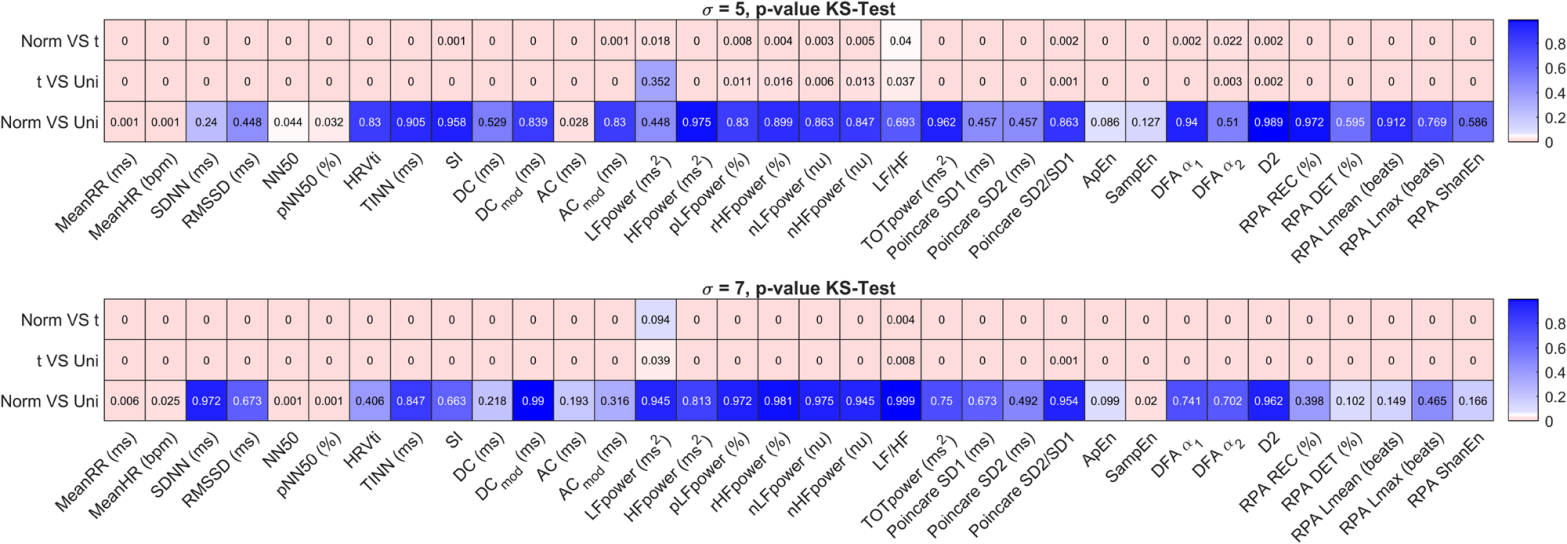
Kolmogorov–Smirnov-test comparing noise of Gaussian distribution, t-distribution, and uniform distribution for two different levels of noise, $\sigma =5$ ms (top graph) and $\sigma =7$ ms (bottom graph). Red color indicates significant difference.

Figure 2 shows the results of the Kolmogorov–Smirnov-test (KS-Test) when comparing Gaussian distribution and t-distribution (first row), t-distribution and uniform distribution (second row), and Gaussian and uniform distribution for noise levels of $\sigma = 5$ ms and $\sigma = 7$ ms.

First, if we assume a p-value of 0.05 to indicate statistical significance, we see that the t-distribution results in significantly different distribution of errors for the majority of HRV metrics when compared to the uniform distribution or the Gaussian distribution. This effect is more pronounced at the higher noise level. At the same time, when comparing Gaussian and uniform distribution, only for very few metrics (MeanRR, NN50, pNN50, and SampEn), a significant difference could be observed at $\sigma =7$ ms. Hence, we will only examine t-distribution and Gaussian distribution in the following.

### Error vs. sigma (mean, 5th percentile and 95th percentile)

**Figure 3:**
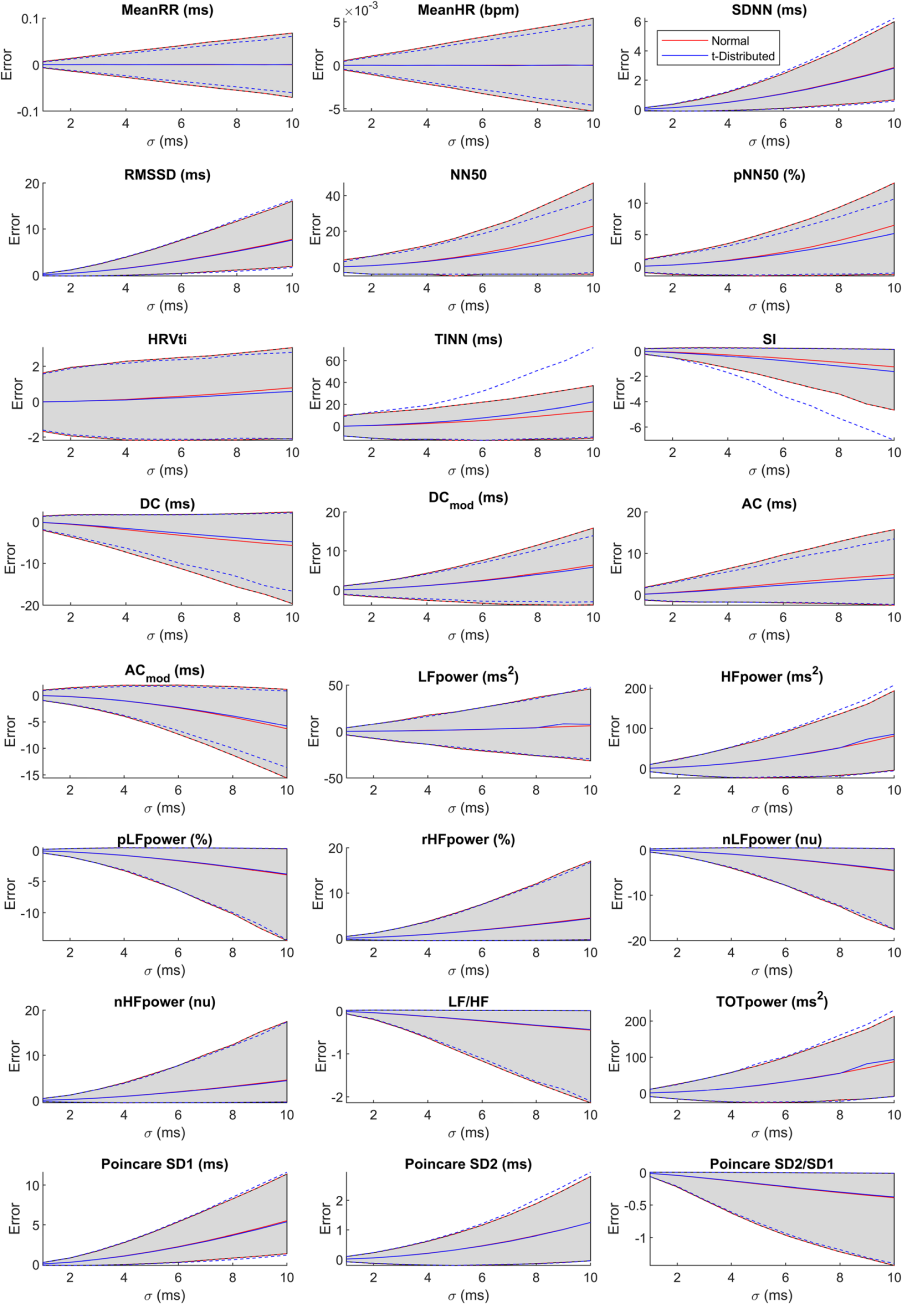

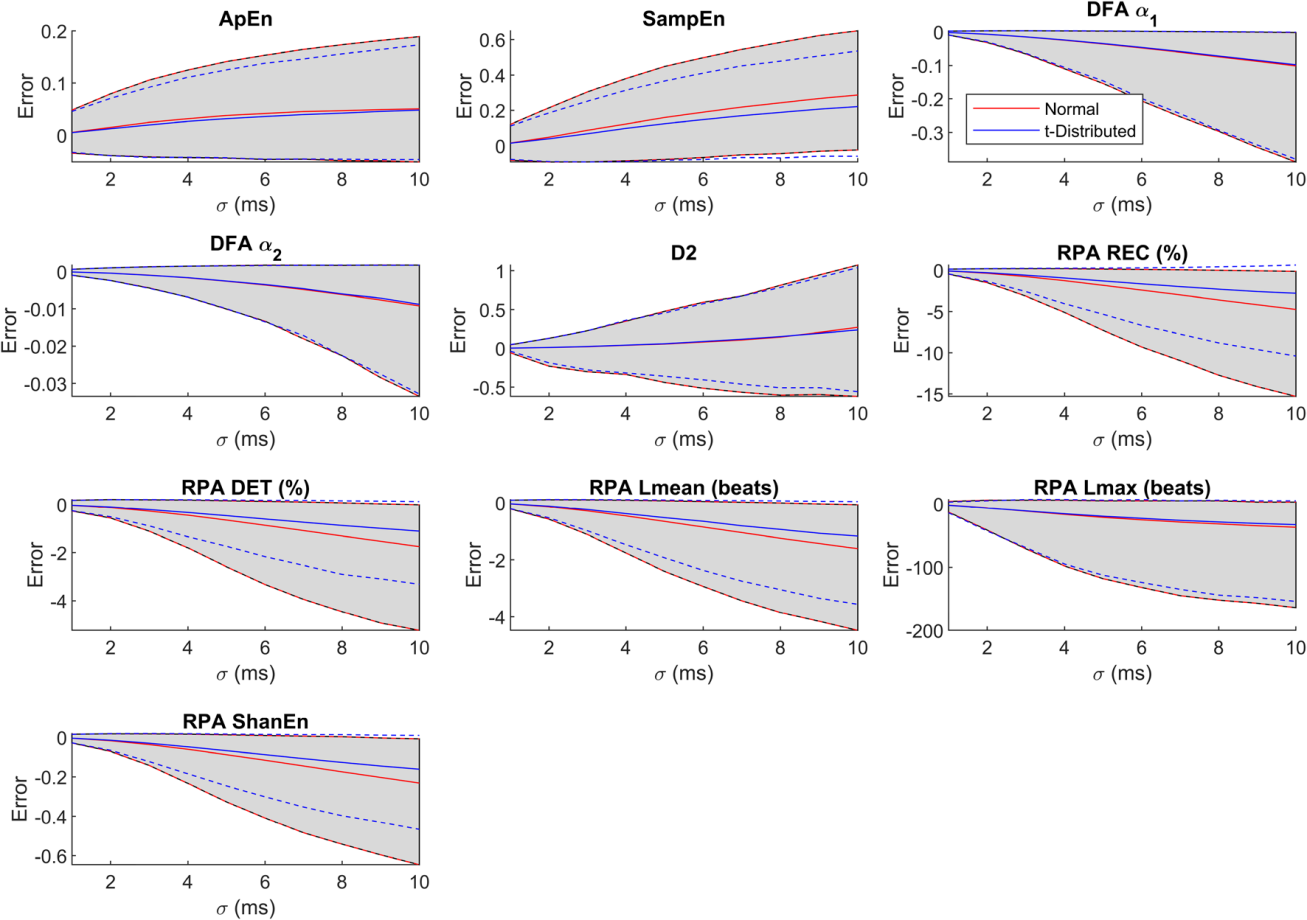
Evolution of the error over the level of noise in terms of mean, 5th, and 95th percentile for all HRV metrics. The blue lines show the evolution for t-distributed noise, the red line for Gaussian noise. The unit of the error is given in the panel title.

In the above Fig. 3, we plot the systematic bias, the 5th percentile and the 95th percentile for all HRV metrics over $\sigma$. The gray shaded area marks the area enclosed by the 95th and 5th percentile when Gaussian noise is added.

As expected, all parameters show an increase in absolute error as indicated by the shaded area with an increase in $\sigma$. Additionally, with an increase in $\sigma$, almost all parameters show an increase in systematic bias with either a positive or a negative sign, i.e. almost all parameters are either systematically over- or underestimated when heart beat locations are noisy. Unsurprisingly, only the mean heart rate and the mean RR interval do not show a systematic bias. Also, although the KS-test has revealed statistically significant differences in error distribution, when either t-distributed or Gaussian noise is considered, we can only make out small differences for most parameters. However, stark differences are obvious in NN50, pNN50, TINN, SI, and all RPA metrics.

### Dependency of the relative error on $\sigma$

**Figure 4:**
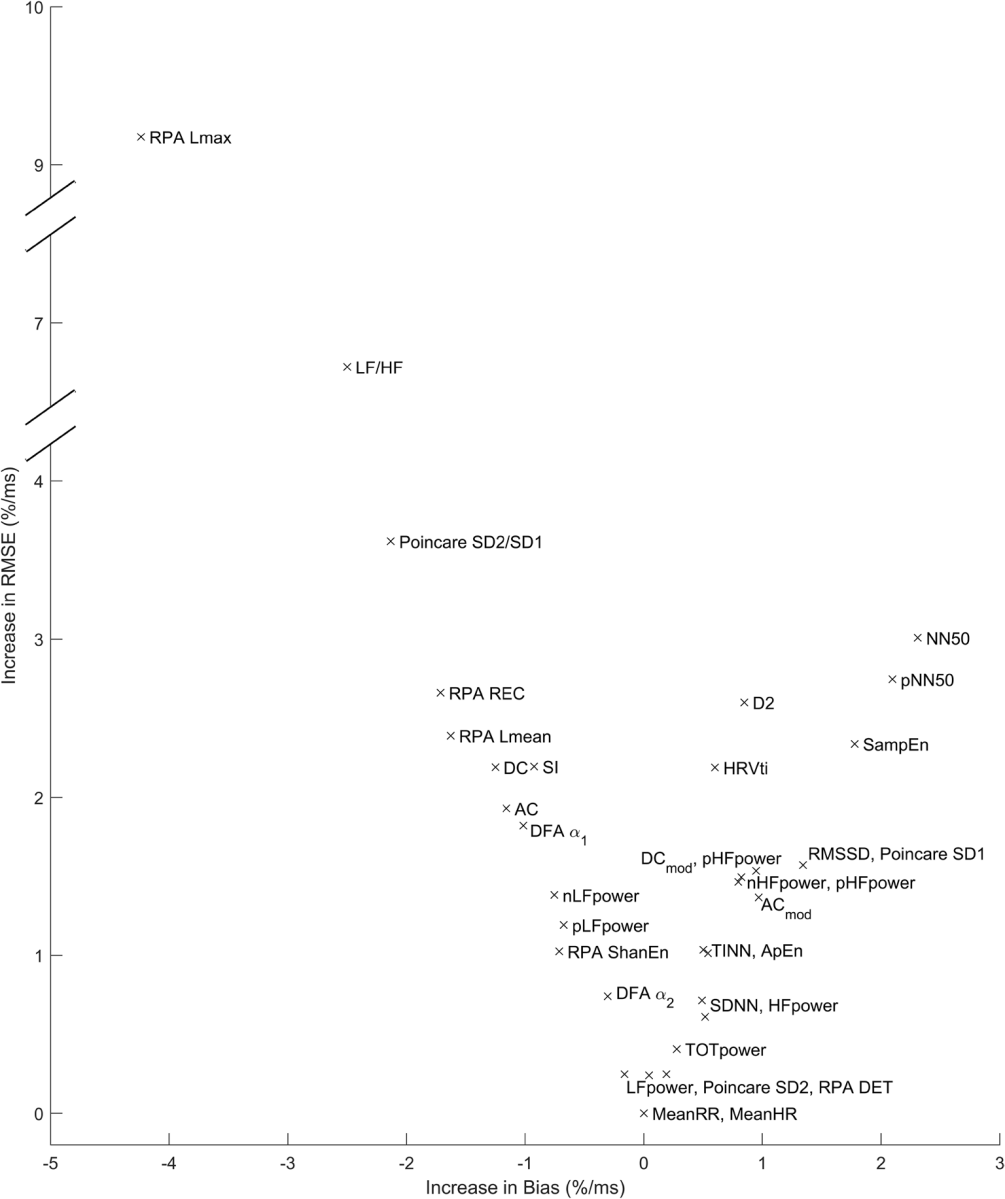
Visualization of $\alpha _{\textrm{RMSE}}$ over $\alpha _{\textrm{bias}}$ for all HRV metrics, see also Table 2.

As the previous analysis has demonstrated, all parameters are influenced in terms of absolute error by the addition of noise, and almost all parameters show an increase in systematic bias. Table 2 shows the distribution of the ground truth data for all HRV metrics in terms of mean, 5th percentile, and 95th percentile. Column 5 to 7 show the results of the linear fit as described in Eqs. (10)–(12), i.e. the linear increase of bias, MAE and RMSE, respectively, in dependency of $\sigma$. The table is sorted in ascending order in terms of $\alpha _{\textrm{RMSE}}$ (see Eq. 12). To visualize the dependency of the normalized error, Fig. 4 shows $\alpha _{\textrm{bias}}$ over $\alpha _{\textrm{RMSE}}$ for all HRV metrics.

**Table 2:** Distribution of all HRV metrics in terms of mean, 5th, and 95th percentile as well as the linear fitting factors $\alpha _{\textrm{bias}}$, $\alpha _{\textrm{MABS}}$, and $\alpha _{\textrm{RMSE}}$ with respective Pearson correlation *r* for the goodness of the linear fit. The values are sorted by $\alpha _{\textrm{RMSE}}$ in ascending order, see also Fig. 4. Interesting observations are printed in bold.

Parameter	Group mean	5th prct.	95th prct.	Linear factor $\alpha$ (*r*) for		
				Bias	MABS	RMSE
MeanHR (bpm)	68.85	54.50	85.91	0.00 (0.28)	0.00 (1.00)	0.00 (1.00)
MeanRR (ms)	888.20	698.38	1101.00	− 0.00 (− 0.41)	0.00 (1.00)	0.00 (1.00)
**LFpower** (ms^2^)	**1119.17**	**86.50**	**3736.62**	**0.04 (0.96)**	**0.15 (1.00)**	**0.24 (1.00)**
Poincare SD2 (ms)	52.09	17.50	110.15	0.19 (0.97)	0.20 (0.97)	0.25 (0.98)
RPA DET (%)	96.73	94.42	98.82	− 0.16 (− 0.99)	0.17 (0.99)	0.25 (1.00)
TOTpower (ms^2^)	2490.91	176.98	8844.93	0.28 (0.96)	0.32 (0.98)	0.41 (0.99)
SDNN (ms)	44.43	13.87	97.98	0.52 (0.97)	0.52 (0.97)	0.61 (0.97)
HFpower (ms^2^)	1300.68	44.50	4740.47	0.49 (0.96)	0.56 (0.98)	0.71 (0.99)
DFA $\alpha _2$	0.25	0.09	0.46	− 0.30 (− 0.97)	0.33 (0.98)	0.74 (0.99)
ApEn	1.10	0.95	1.24	0.54 (0.96)	0.73 (0.94)	1.01 (0.94)
RPA ShanEn	2.94	2.59	3.36	− 0.71 (− 0.99)	0.73 (0.99)	1.02 (1.00)
TINN (ms)	224.38	71.00	469.50	0.50 (0.97)	0.77 (0.99)	1.04 (0.93)
pLFpower (%)	48.79	19.65	77.89	− 0.67 (− 0.98)	0.71 (0.98)	1.19 (0.99)
AC_mod_ (ms)	− 51.82	− 119.81	− 11.51	0.97 (0.97)	1.11 (0.98)	1.37 (0.98)
**nLFpower** (nu)	51.64	20.14	83.88	− **0.75** (− **0.98**)	0.79 (0.98)	1.38 (0.99)
**nHFpower** (nu)	48.31	16.09	79.82	**0.80 (0.98)**	0.83 (0.98)	1.46 (0.99)
pHFpower (%)	46.23	14.87	78.08	0.82 (0.98)	0.86 (0.98)	1.49 (0.99)
DC_mod_ (ms)	54.96	11.12	147.21	0.95 (0.97)	1.25 (0.99)	1.53 (0.99)
Poincare SD1 (ms)	33.95	7.42	84.15	1.34 (0.98)	1.35 (0.98)	1.57 (0.98)
RMSSD (ms)	47.94	10.48	118.82	1.34 (0.98)	1.35 (0.98)	1.57 (0.98)
DFA $\alpha _1$	0.88	0.49	1.32	− 1.01 (− 0.99)	1.02 (0.99)	1.82 (1.00)
AC (ms)	− 40.87	− 94.96	− 8.60	− 1.16 (− 1.00)	1.38 (1.00)	1.93 (1.00)
HRVti	10.55	4.07	19.80	0.60 (0.97)	1.62 (0.90)	2.19 (0.83)
DC (ms)	44.22	8.65	113.89	− 1.25 (− 1.00)	1.43 (1.00)	2.19 (1.00)
SI	11.86	4.65	23.34	− 0.92 (− 0.99)	1.00 (0.99)	2.19 (1.00)
**SampEn**	**1.68**	**1.22**	**2.05**	**1.78 (1.00)**	**1.91 (0.99)**	**2.34 (0.99)**
RPA Lmean (beats)	9.19	6.57	13.18	− 1.63 (− 0.99)	1.65 (0.99)	2.39 (1.00)
D2	2.29	0.01	4.22	0.85 (0.94)	1.76 (0.99)	2.60 (1.00)
RPA REC (%)	25.26	16.13	39.01	− 1.71 (− 0.99)	1.73 (0.99)	2.66 (1.00)
**pNN50** (%)	**23.94**	**0.00**	**67.19**	**2.10 (0.96)**	**2.31 (0.97)**	**2.75 (0.97)**
**NN50**	**76.06**	**0.00**	**202.00**	**2.31 (0.96)**	**2.51 (0.96)**	**3.01 (0.97)**
Poincare SD2/SD1	1.77	0.98	2.86	− 2.13 (− 1.00)	2.14 (1.00)	3.62 (1.00)
**LF/HF**	**1.67**	**0.25**	**5.22**	− **2.50** (− **0.99**)	**2.54 (1.00)**	**6.72 (1.00)**
RPA Lmax (beats)	92.54	38.00	227.00	− 4.24 (− 0.99)	4.50 (0.99)	9.18 (0.95)

Several interesting observations can be made. First, the sensitivity of the parameters varies quite dramatically, ranging from close to 0 to almost 9% per ms. As expected, SDNN is very robust, as an increase of the power of the noise by 1 ms will increase the RMSE by 0.6% relative to the group mean. This is even more pronounced, with absolute LF power (0.2% RMSE per ms). Particularly problematic is the ratio LF/HF, which is a commonly used HRV parameter to assess sympathovagal balance, but exhibits strong underestimation as well as a large RMSE. This is consistent with the observation that normalized LF power is underestimated, while normalized HF power is overestimated. Interestingly, Sample Entropy is also quite sensitive ($\sim$ 2%/ms for Bias/MABS/RMSE). Similarly, although often used, pNN50 and NN50 show overestimation in the range of 2%/ms and an increase in RMSE in the range of 3%/ms.

### Bland–Altman analysis

**Figure 5:**
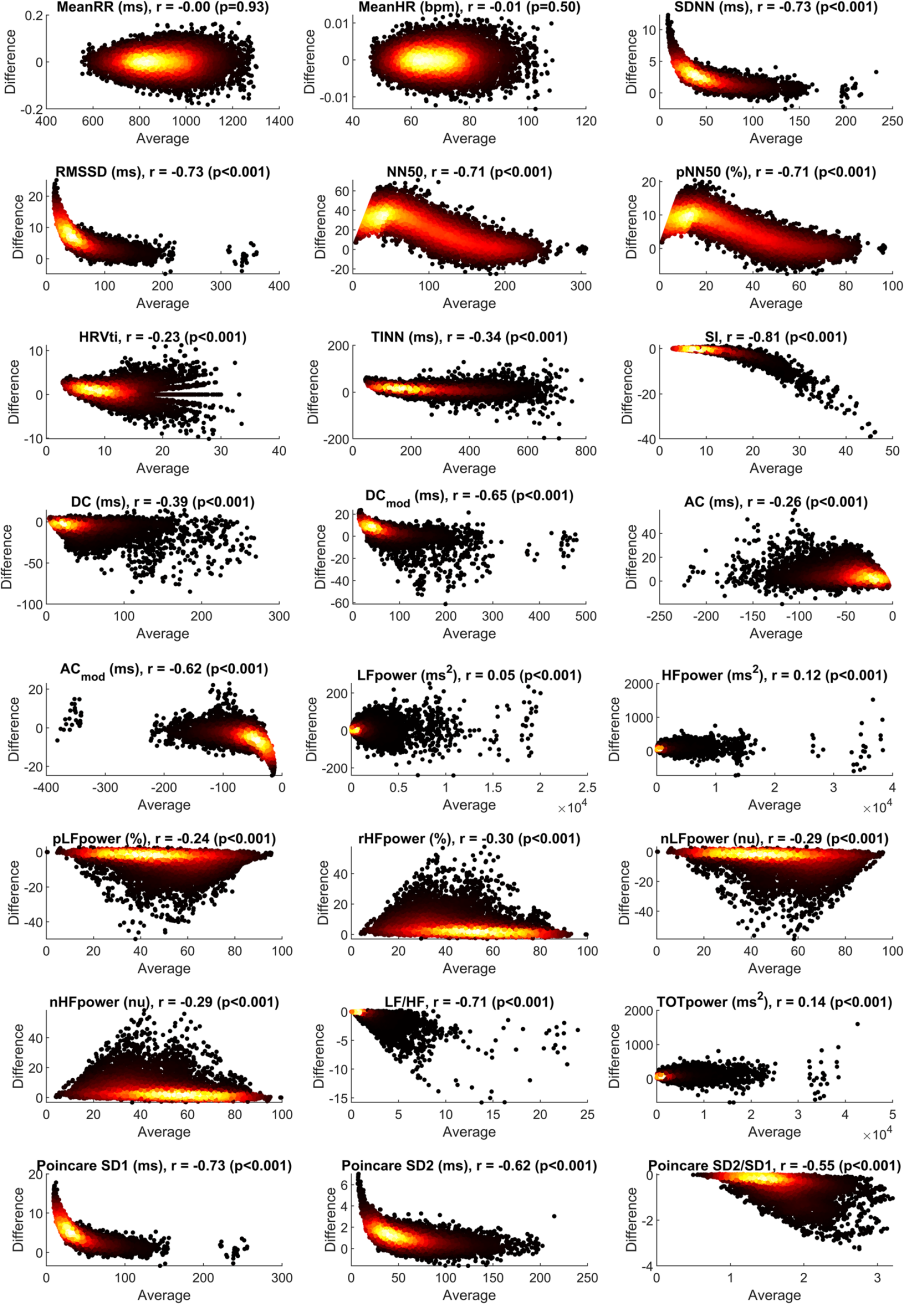

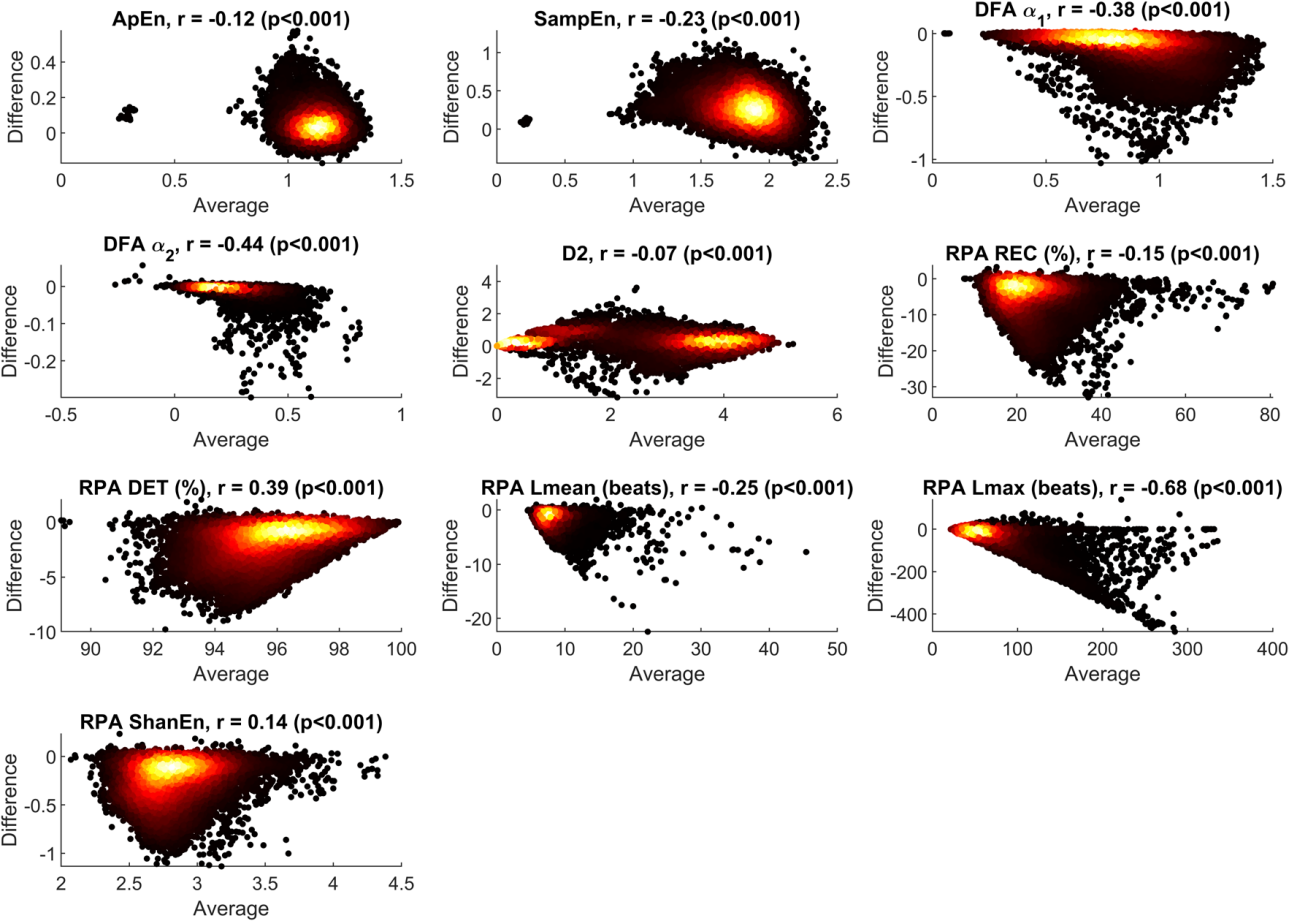
Bland–Altman plots for all HRV metrics (Gaussian noise, $\sigma =10$). To avoid clutter, the density is color-coded, i.e. individual points are darker, points with many neighbouring points are brighter.

We have seen in the previous section that the noise may create a systematic bias. It remains to be analyzed if this bias is independent of the actual value of the parameter. If that were the case, the Bland–Altman (BA) plots would be point clouds symmetric to a line parallel to the y-axis. In the following, we present the BA plots for a noise level of $\sigma = 10$ and consider Gaussian noise. Moreover, we give the Pearson correlation coefficient *r* of average $a_{i,p}$ (x-axis) and difference $e_{i,p}$ (y-axis). To avoid clutter, usually a small random subset of the data is used for plotting in the standard BA plot. However, we introduce color-coding for the individual points to give information about the point density (Fig. 5).

## Discussion

We know from system theory that a linear operation applied to Gaussian noise will result in Gaussian noise, and only its mean and standard deviation may be altered. For the general case of nonlinear operations and other distributions of noise, no straight-forward general description can be given. Instead, an extensive Monte Carlo - type analysis as the one presented here can be used. In this mathematical sense, almost all HRV metrics analyzed here involve non-linear calculations. Hence, it is not surprising that we can see from all figures and tables that the commonly used HRV metrics show great variability when it comes to their behaviour under artificially added noise.

From Fig. 2 we can see that the effect of the distribution of the noise on the HRV metrics when using either uniform noise or Gaussian noise are mostly negligible, with the exception of NN50 and pNN50. As argued above, uniform noise applied to the location of individual heart beats will result in a triangular noise distribution in the intervals. A visual inspection of Fig. 1 shows the similarity of the triangular distribution and the Gaussian distribution, and makes this result intuitively plausible. We do however learn that the heavy-tailed t-distribution will result in significant differences for almost all parameters. Again, this is plausible as the t-distribution has a higher probability of generating outliers compared to the Gaussian distribution (and obviously the uniform distribution). Still, statistical significance does not give information about the effect size. Hence, if we look at Fig. 1, we learn that for most parameters, the distribution of the error is very similar when Gaussian or t-distributed noise is added. However, notable exceptions such as, for example, TINN, NN50, pNN50, and SampEn do exist. Hence, we would argue that as a first step, analyzing an HRV metric’s sensitivity towards noise can be achieved using Gaussian noise. However, for an in-depth analysis, it makes sense to determine the actual distribution of noise generated by the measurement system (measurement modality and beat detection algorithm) to be used, and quantify its impact on the HRV metric in question.

We can see from the BA plots in Fig. 5 that in addition to an offset, a systematic error is introduced for most parameters with very few exceptions. Again, the effect varies from parameter to parameter. For example, for SDNN and RMSSD, small values are systematically over-estimated as shown by $r \approx 0.8$. Note that we learned from Table 2 that in general, the bias introduced by noise is relatively small for SDNN, emphasizing that the main source of over-estimation stems from comparatively small absolute parameter values. On the other hand, we could also see in Table 2 that, for example, absolute HF power is systematically over-estimated with a linear factor approximately close to SDNN. But we can see from the BA plot that this over-estimation is manifested in a relatively constant offset and is not correlated with the ground truth value of absolute HF power. No correlation is also found for absolute LF power and total power. Again, this is plausible as the addition of noise will always result in an addition of signal energy which is measured by these parameters. A common strategy for compensating uncertainty is to use averaging over several segments for which HRV is calculated. However, due to the significant bias in most of the parameters, averaging is limited as a tool for error reduction. The *relative* power metrics show a different behavior. This can be explained since additive noise on beat locations will particularly influence the higher frequencies of beat-to-beat intervals as they are calculated via differentiation, which amplifies high frequencies. Generally, HRV parameters that reflect higher frequencies perform poorer with respect to Bias and RMSE. Besides the differentiation, the noise applied is local and statistically independent between different time points, which reflects the type of errors expected from the processing pipeline. Notably, lower frequency errors that are dependent over multiple beats can appear for specific choices of algorithms and modalities (e.g. respiratory amplitude modulation in PPG) as described in^16^. In conclusion, we believe that it is important for HRV analysis in general to closely examine the distribution of the parameters instead of comparing only mean/median values. Also, one has to keep in mind that different cohorts may have different baseline distributions and hence the impact of noise may be an important factor.

In terms of individual parameters, several interesting observations were made. For example, NN50/pNN50 show inferior results in terms of bias, RMSE, sensitivity to the distribution of the noise, and dependency of the influence of noise on the ground truth value (Fig. 5). These findings align with studies that found pNN50 to be sensitive to missing beats^14^. Hence, we would argue that this simple, seemingly robust parameter should be used with caution, in particular when the baseline values are expected to be low (e.g. in older subjects and patients under physiological stress e.g. due to infection). Another “underperformer” is the LF/HF ratio. It can be expected that this parameter is very sensitive to noise as its derivation involves the calculation of a ratio of values, which are themselves sensitive to noise. Nevertheless, we find it somewhat surprising how poorly this parameter performs in terms of bias and absolute error/RMSE compared to the other parameters (Fig. 4). LF/HF is a popular parameter and we have used it in our previous works as well. In light of our current findings, we suggest to use extreme caution when using LF/HF in future studies, as small differences in noise in the groups to be distinguished may result in severe differences that may have nothing to do with the underlying physiology. The LF power in normalized units is less sensitive to noise and should be preferred over the LF/HF in studies which wish to use the frequency-domain analysis of HRV to assess sympathovagal balance of the autonomic nervous system.

In Table 2 we present the ground truth distribution of the HRV metrics we analyze. From the values and the fact that the used “Autonomic Aging”-dataset contains data from a population of young to old individuals of both sexes, we believe we cover a reasonable range of values and our results should generalize well. However, one hallmark of the database is that the subjects were “rigorously screened” to ensure healthiness and that the measurements are performed in a resting condition while maintaining wakefulness. Hence, different results may be obtained if data from severely diseased and/or non-resting individuals are used, provided their baseline values fall completely out of the ranges analyzed here. Hence, to make sure our results are applicable to a certain study, one should first check if the obtained values fall within the ranges of the present study. For certain HRV parameters (especially frequency domain variables) implementation details might also change the results slightly and pre-processing of the intervals plays a large role.

Let us emphasize again that to make the influence of noise on RMSE and bias comparable, one has to employ some sort of normalization. In this case, the error was normalized by the population mean (see Table 2) of a broad population. Additionally, our Bland–Altman analysis shows that the over- or underestimation of parameters may depend heavily on the ground truth value (e.g., small SDNN values may be over-over-estimated). As a consequence, the sorting of the HRV metrics in Table 2 may differ if a specific population is analyzed. For instance, the population mean of SDNN was found to be 44.43 ms, the one of HFpower to be 1300.68 ms^2^ for this cohort of more than 1000 subjects. When for instance analyzing the much smaller Fantasia-dataset (20 subjects 20–34 years and 20 subjects 68–85 years), SDNN was similar (39.56 ms), while HFpower was on average almost half of the value of the large cohort (702.47 ms^2^). Hence, with respect to this population mean, HFpower would be twice as sensitive. At the same time, note that the population mean only affects the normalized comparison. Still, even on the small dataset we observed the same general tendencies (small errors for MeanHR/RR and SDNN, larger errors for HF parameters compared to LF parameters, worst performance for RPA Lmax, NN50, LF/HF).

In order to evaluate in which error-range a given sensor and method *M* falls we suggest the following procedure: Take multiple simultaneous measurements with the test-device and a validated ECG-patient-monitor with sampling frequency of 1000 Hz or more for 5 min and at least 10 participants and varying age groups. Then compute the reference intervals with a validated QRS detector and verification of a trained Cardiologist. Subtract the reference intervals from the intervals estimated by *M* and estimate the standard deviation. Based on the standard deviation and the here presented reference values it can be checked if certain HRV parameters might be good enough.

## Conclusion

In this paper we analysed the effect of uncertainty in temporal heartbeat location in various HRV parameters using a Monte Carlo simulation approach. The results showed that there are large differences between the HRV parameters in the robustness against errors in the heartbeat interval tachogram. The least tolerant ones being HF/LF ratio, pNN50 and NN50 and the most tolerant being RPA DET, LFpower but also SDNN. Three common noise distributions were evaluated in this study where the error range was limited to relatively small values and the influence of systematical errors over multiple beats was neglected. Future research should evaluate particularly the noise profiles (distributions and amplitudes) commonly seen in novel measurement modalities other than ECG, e.g., PPG, remote imaging PPG, seismo- and ballistocardiogram that commonly have larger uncertainty and/or might be influenced by systematic errors due to physiology. Biases in HRV parameters such as SDNN could possibly be partially compensated algorithmically if the noise distribution is known. On the other hand, simple averaging of HRV parameters over several analysis segments does not account for systematic bias. Most importantly, researchers should be cautious about the robustness of different HRV parameters to noise in the beat-to-beat interval data. To bring HRV closer to clinical practice, recommendations specific to application should be developed based on our findings.

## Data Availability

Raw data is available at https://physionet.org/. Derived data supporting the findings of this study are available from the corresponding author [M.R.] on request.
